# Sensitivity of Physiological Emotional Measures to Odors Depends on the Product and the Pleasantness Ranges Used

**DOI:** 10.3389/fpsyg.2015.01821

**Published:** 2015-12-01

**Authors:** Aline M. Pichon, Géraldine Coppin, Isabelle Cayeux, Christelle Porcherot, David Sander, Sylvain Delplanque

**Affiliations:** ^1^Swiss Center for Affective Sciences, University of GenevaGeneva, Switzerland; ^2^Emotion, Elicitation and Expression Laboratory, Department of Psychology, Faculté de Psychologie et des Sciences de l’Éducation, University of GenevaGeneva, Switzerland; ^3^Firmenich S.A.Geneva, Switzerland

**Keywords:** odor perception, emotion, psychophysiology, pleasantness, subjective sensitivity, physiological sensitivity, fragrance

## Abstract

Emotions are characterized by synchronized changes in several components of an organism. Among them, physiological variations provide energy support for the expression of approach/avoid action tendencies induced by relevant stimuli, while self-reported subjective pleasantness feelings integrate all other emotional components and are plastic. Consequently, emotional responses evoked by odors should be highly differentiated when they are linked to different functions of olfaction (e.g., avoiding environmental hazards). As this differentiation has been observed for contrasted odors (very pleasant or unpleasant), we questioned whether subjective and physiological emotional response indicators could still disentangle subtle affective variations when no clear functional distinction is made (mildly pleasant or unpleasant fragrances). Here, we compared the sensitivity of behavioral and physiological [respiration, skin conductance, facial electromyography (EMG), and heart rate] indicators in differentiating odor-elicited emotions in two situations: when a wide range of odor families was presented (e.g., fruity, animal), covering different functional meanings; or in response to a restricted range of products in one particular family (fragrances). Results show clear differences in physiological indicators to odors that display a wide range of reported pleasantness, but these differences almost entirely vanish when fragrances are used even though their subjective pleasantness still differed. Taken together, these results provide valuable information concerning the ability of classic verbal and psychophysiological measures to investigate subtle differences in emotional reactions to a restricted range of similar olfactory stimuli.

## Introduction

Olfaction stands out in the sensory landscape for its peculiar and intimate connection with the world of emotions, which may stem from the distinctive anatomical overlap between olfactory- and emotion-related neural structures (Carmichael et al., 1994; Smeets and Dalton, 2002; Anderson et al., 2003; Grabenhorst et al., 2007; Zelano et al., 2007). The majority of consciously perceived odors tend to be salient, compared with stimuli from other modalities, because of the prominent presence of their hedonic dimension (Mohanty and Gottfried, 2013). Odors surround us in everyday life and affect our behavior (Bensafi et al., 2002a; Li et al., 2007), our mood, and our well-being (Alaoui-Ismaïli et al., 1997; Rétiveau et al., 2004; Warrenburg, 2005. This is attested by the importance of perfumery since the earliest civilization (Le Guérer, 1994), the significantly impoverished quality of life observed in individuals suffering from olfactory impairment (Hummel and Nordin, 2005; Landis et al., 2009; Croy et al., 2012; Keller and Malaspina, 2013), and the influence that odors exert on various behavioral and cognitive processes such as memory or preference acquisition (Leppanen and Hietanen, 2003; Herz et al., 2004a).

Emotions are characterized by synchronized changes in several components of the organism: subjective, physiological, expressive, cognitive, and motivational (Scherer, 1982, 2001). Experimental research using olfactory stimulations has demonstrated changes in these components as a function of odor pleasantness. At the subjective level, self-reports (e.g., on liking scales) are used extensively to characterize individual preferences (Degel et al., 2001; Savic et al., 2002; Howard et al., 2009; Pause et al., 2009; Adolph et al., 2010; Li et al., 2010; Gelstein et al., 2011; Coppin et al., 2012). Self-reported measures of preference are deeply influenced by contextual factors and individual states, as the subjective response to smell is notoriously flexible (see Coppin and Sander, 2011 for a review). Hedonic responses to olfactory stimulations can be modulated by processes such as mere exposure (Delplanque et al., 2008, 2015), decision making (Coppin et al., 2010), associative learning (Herz et al., 2004b), or verbal context (Herz, 2003; Bensafi et al., 2007). According to appraisal theories, including the component process model (Scherer, 1982, 2001), the physiological component of the emotional response is a support for adapted responses and energy that provides for the expression of these action tendencies. Extensive experimental evidence shows that olfactory stimulations induce differential responses at the physiological level according to their pleasantness, readily affecting heart rate, which has been shown to decrease as a function of odor hedonicity (Alaoui-Ismaïli et al., 1997; Bensafi et al., 2002b; Delplanque et al., 2009), while other indicators such as skin conductance and pupillary light reflex are also sensitive to arousal (Bensafi et al., 2002b; Bradley et al., 2008; Sequeira et al., 2009). Finally, the expressive component of the emotional response is subtended by the motor system and is responsible for communication of reaction and behavioral intention. Odor pleasantness also affects facial expression, inducing differences in EMG activity. Facial muscles responsible for frowning (corrugator) and for smiling (zygomaticus) respond differentially to pleasant and unpleasant odors (e.g., Bensafi et al., 2002c; Soussignan et al., 2005; Armstrong et al., 2007; Delplanque et al., 2009).

Most previous experiments have used varied olfactory stimuli, spanning a wide valence spectrum (i.e., very unpleasant to very pleasant; see Mohanty and Gottfried, 2013 for a review), which increases the likelihood of observing clear-cut differences in all components of the emotional response. A comparison between physiological and self-reported responses to olfactory stimulations (Alaoui-Ismaïli et al., 1997) has revealed that the correlation between these two indicators is good, as long as the stimulations are well contrasted in terms of subjectively reported valence and are of different types (e.g., food, cosmetics, animal). Certain types of odors, such as essential oils or fine perfumes, can be considered as belonging to one particular odor family—fragrances—in which marked differences in self-reported pleasantness can nonetheless be observed (Rétiveau et al., 2004).

Subjective reports appear to provide subtle valence differences that are found even when the odors belong to the same family. Subjective feelings integrate all other emotional components and are plastic (Scherer, 1982, 2001). By contrast, the physiological component supports adapted responses and energy, providing for the expression of more hard-wired action tendencies. This component is less likely than subjective feelings to be able to differentiate subtle differences in valence for odors of the same family. Here, we illustrate this point by presenting the results of two studies that assess subjective, physiological, and expressive components of emotion in response to olfactory stimuli. We compared two conditions: (1) *Odors*: when olfactory stimulations were strongly differentiated and belonged to different odor families (food, floral, animal, perfumes, etc.), and (2) *Fragrances*: when olfactory stimulations belonged to a particular family, i.e., fine perfumes. The objectives of this study were (1) to replicate the classic distinction observed in emotional components (subjective, physiological, and expressive) in response to well-differentiated olfactory stimulations (i.e., pleasant and unpleasant odors); and (2) to evaluate whether these components remain sensitive enough to differentiate between the emotional reactions associated with family related olfactory stimulations (i.e., fragrances). If indeed the subjective component is more malleable than the physiological component, then subjective differences should arise regardless of width of the pleasantness spectrum examined, whereas physiological differences would appear only in the case of larger differences.

Because olfactory preferences are highly individual, we did not contrast the different dependent variables (i.e., subjective, physiological, and expressive) by olfactory stimuli, but performed individual selections, grouping each individual’s most pleasant and most unpleasant olfactory stimuli.

## Materials and Methods

We analyzed non-published data acquired previously by Delplanque et al. (2009). In this study, participants were presented with a set of varied “sample” and “target” odors and given no information about them. Sample odors were presented first as an encoding condition, whereas target odors were presented second, as a retrieval condition. Only target odors were previously analyzed to be included in Delplanque et al. (2009). Here, we analyzed responses to the sample odors. Emotional responses to these odors were compared with those obtained in an independent sample of participants presented with a set of fragrances. Given the strong inter-individual variability of olfactory preferences (Herz and Von Clef, 2001; de Araujo et al., 2005; Keller et al., 2007), we conducted our analyses on the basis of individual judgments as opposed to averaging the subjective ratings for a given odor.

### Participants

Two different groups of nonsmoking participants (Group 1 and Group 2), all University of Geneva students, were recruited through ads posted in a university building. Group 1 consisted of 18 participants (9 females, right handed; mean age = 27.1 ± 6.2 years) and was provided with pleasant and unpleasant odors (Delplanque et al., 2009). Group 2 consisted of 21 participants (all females; mean age = 22.7 ± 3.3 years) and was provided with fragrances. Participants were individually tested and paid 50 Swiss Francs (approximately $50) for their participation. On testing days, participants were asked not to wear any perfume. They all self-reported a normal sense of smell and were free from respiratory infections when they participated. None of the participants reported any mental illnesses that could have affected their emotional responses to stimuli. Written consent was obtained from all participants before starting the experiment in accordance with the Declaration of Helsinki, and the study was approved by the ethical committees of the Geneva University Hospital and of the Psychology Department of the University of Geneva. In Group 1, two participants were excluded because of acquisition artifacts in facial muscle activity (both the corrugator and the zygomaticus muscles), leaving 19 participants for analysis. In Group 2, participants were excluded because of acquisition artifacts in activities of the corrugator (one participant) and zygomaticus muscles (two participants), leaving 16 and 17 participants for analysis on these two variables, respectively.

### Stimuli

All olfactory stimuli (“*Odors*” and “*Fragrances*”) were injected into the tampon of cylindric felt-tip pens (14 cm long, inner diameter 1.3 cm). The use of these highly practical devices (provided by Burghart, Germany) avoids any contamination of the environment.

#### Odors

Thirty-two *a priori* pleasant and unpleasant odorants (**Table 1**) were selected on the basis of a previous study conducted on 66 participants, who evaluated 51 odorants according to subjective intensity, pleasantness, and familiarity (see Delplanque et al., 2008, 2009). The aim of this large selection was to obtain an array of odorants with a wide pleasantness spectrum. For practical reasons, we labeled this first choice of odorants as “*Odors*”.

**Table 1:** Odors.

Unpleasant odors	Concentration (% in DIPG)	Odor family	CAS	Pleasant odors	Odor family	Concentration (% in DIPG)	CAS
Aladinate^∗^	20	Floral	341017-24-1	Amyl acetate^∗^	Fruity	20	628-63-7
Beer	20	Savory food		Basil	Green	5	
Body odor (synthetic)	Pure	Animal		Bornyl acetate^∗^	Camphor	20	125-12-2
Carbinol^∗^	5	Earthy	700-06-1	Cake	Sweet food	20	
Caproic acid^∗^	20	Animal	142-52-1	Cassis bud	Fruity	20	
Diacetyl^∗^	50	Buttery	431-03-8	Classic body lotion fragrance	Detergent	5	
Durian	20	Fruity		Classic detergent fragrance	Detergent	1	
Dynascone^∗^	20	Amber, Musky	0056973-85-4	Classic shampoo fragrance	Detergent	10	
Framboisone^∗^	50	Fruity		Classic soap fragrance	Detergent	10	
Ghee	5	Savory food		Fig	Fruity	10	
Isobutyl quinoline^∗^	20	Animal	93-19-6	Geraniol^∗^	Floral	20	106-24-1
Isobutyric acid^∗^	10	Pungent, Animal	79-31-2	Green tea	Floral green	10	
Isovaleric acid^∗^	1	Pungent, Animal	503-74-2	Honey	Sweet food	10	
Landes wood	5	Woody		Lavender	Floral	10	
Leather	5	Animal		Lilac	Floral	10	
Melonal^∗^	50	Fruity	106-72-9	Lime	Citrus	20	
Octamylamine^∗^	5	Fishy-oily	502-59-0	Linalol^∗^	Floral	10	78-70-6
Octanol^∗^	5	Oily	11-87-5	Magnolia grandifolia	Floral	20	
Paracresol^∗^	1	Animalic	106-44-5	Methyl-salicylate^∗^	Aromatic	10	119-36-8
Rancid butter	20	Savory food		Neroli	Floral	5	
Sclarymol^∗^	1	Sulfury		Peach	Fruity	10	
Skunk	10	Animal		Pineapple	Fruity	10	
Sulfox	0.05	Sulfury		Tiare	Floral	Pure	
Yogurt	10	Sweet food		Tutti frutti	Fruity	10	

*^∗^Single odorant molecule. CAS molecule numbers are provided where available.*

#### Fragrances

Nine additional fine perfumes (**Table 2**) were selected on the basis of a preliminary study performed on 60 undergraduate students (60 females; mean age = 20.27 ± 3.1 years). The primary interest of that study was to assess the influence of contextual information on fragrance evaluation. We chose fragrances that were well-known in the French and Swiss markets. In addition, the fragrances were well characterized to ensure good perceptual variability (see **Table 2**). For practical reasons, we labeled this second choice of odorants as “*Fragrances*”.

**Table 2:** Fragrances.

Fragrance	Brand	Notes
Angel	Thierry Mugler	Oriental – Vanilla
Chanel n°5	Chanel	Floral – Aldehyde
Ck One	Calvin Klein	Citrus – Aromatic
Flower	Kenzo	Cedarwood – Amber – Musks
J’adore	Dior	Floral – Fruity
Light blue	Dolce & Gabbana	Floral – Fruity
Romance	Ralph Lauren	Floral – Fruity
Samsara	Guerlain	Oriental – Woody
Trésor	Lancôme	Floral – Rose Violet

### Experimental Procedures

Participants were told that they would be provided with olfactory stimuli to evaluate. During one session, they smelled the 32 odor-containing (Group 1, *Odors*) or the nine fragrance-containing (Group 2, *Fragrances*) pens in random order in successive trials. For each trial, an experimenter seated near the participant in a well-ventilated room then placed an odor pen about 1 cm below the participant’s nostrils for 2 s. Before testing, participants were instructed via computer to smell the odorants according to a particular procedure to minimize variability in intra- and inter-participant breathing patterns (Jung et al., 2006; Delplanque et al., 2009). The participants first had to breathe out deeply through the mouth, wait for the request to inhale (a word presented on a screen in front of the participant), breathe in evenly with the felt-tip pen containing the odorant under the two nostrils, and then rest and relax for 15 s.

The presentation of the olfactory stimulus to the participant was followed by the completion of subjective ratings assessing intensity, hedonicity, and familiarity. The interval between two stimuli was 15 s to avoid sensory adaptation.

### Subjective Ratings

Participants rated the hedonicity, intensity, and familiarity of the olfactory stimuli that they were presented with on continuous 10 cm scales from *very unpleasant* (left of the scale = 0 cm) to *neutral* (middle of the scale, 5 cm) to *very pleasant* (right of the scale, 10 cm); from *not perceived* (or low intensity, left) to *medium* (middle) to *strong* (or high intensity, right); and from *not familiar at all* (left) to *very familiar* (right), respectively, (see Delplanque et al., 2009 for details).

### Apparatus and Physiological Recordings

Physiological signals were assessed with the TEL 100 Remote Monitoring System (Group 1) and the MP150 (Group 2) system of Biopac (Santa Barbara, CA, USA) with separate settings for the electrocardiogram, electrodermal activity, and respiratory activities. Signals were transferred from the experimental room to the MP100 Acquisition Unit (16 bit A/D conversion) in an adjacent room and stored on computer hard disk (sampling rate 500 Hz). Respiratory activity was assessed by placing two respiration belts on the participant that measured abdominal and thoracic expansion and contraction. Electrodermal activity was recorded (high-pass filter: 0.025 Hz) by the constant-voltage method (0.5 V). Beckman Ag–AgCl electrodes (8 mm diameter active area) filled with a skin conductance paste (Biopac) were attached to the palmar side of the middle phalanges of the second and third fingers of the participants’ non-dominant hand. Heart rate was assessed by fixing Biopac pregelled disposable electrodes under the participants’ left and right wrists. A third electrode was placed on the left ankle. The signal was amplified by 1,000 and low-pass filtered (30 Hz). Electrocardiographic R waves were detected oﬄine, and intervals between heartbeats were converted into heart rate, expressed in beats per minute (BPM). Surface electromyography (EMG) was collected, digitized, and stored (bandwidth 0.1 to 417 Hz, sample rate: 2,048 Hz) with a BIOSEMI Active-Two amplifier system (BioSemi Biomedical Instrumentation, Amsterdam, the Netherlands). Six active electrodes were placed over the right frontalis, corrugator, and zygomaticus regions of the face, corresponding to three distinct bipolar montages of interest (Fridlung and Cacioppo, 1986). Two additional electrodes placed above the inion (the common mode sense active electrode and the driven right leg passive electrode) were used as recording references and ground electrodes^1^. Conventional bipolar montages were then calculated from electrode pairs for each muscle by subtracting the activity of one electrode placed over the muscle from the activity of the other nearby electrode in Brain Vision Analyzer software (Brain Products, Gilching, Germany). Signals were then filtered with a 20 to 400 Hz band-pass digital filter, rectified, and low-passed filtered below 40 Hz.

### Physiological Data Analyses

#### Respiration Parameters

The voltage amplitude of the inhalation phase during the olfactory stimulus presentation was reported and constitutes the main respiratory control.

#### Electrodermal Activity

Specific skin conductance responses (SCRs) to odors were measured in microSiemens and analyzed oﬄine. They were scored as changes in conductance starting in the -s to 4-s interval after the beginning of inhalation (Dawson et al., 1990). SCRs were square root transformed to normalize the data (Edelberg, 1972).

#### Facial Muscle Activity

Electromyography amplitude during the 1 s before olfactory stimulus presentation served as the baseline. To allow us to examine the temporal profiles of facial EMG for 5 s after inhalation of different olfactory stimuli, we expressed mean EMG amplitudes during subsequent 1 s time intervals as a percentage of the mean amplitude of the baseline. Percentage scores were introduced to standardize the widely differing absolute EMG amplitudes of individual participants and thus enable comparison between individuals and groups (e.g., de Wied et al., 2006).

#### Heart Rate

The biphasic heart response consists of cardiac acceleration peaking at about 3 s followed by a decrease in heart rate, with a minimum reached at about 6 s after the onset of inspiration (see Delplanque et al., 2009). We analyzed the maximum negative variation in the 5 to 8 s window following stimulus presentation (heart rate deceleration) to investigate whether this phase was sensitive to stimulus pleasantness. The heart rate time course during the 10 s before olfactory stimulus presentation served as the baseline. We averaged the heart rate values within successive 200 ms periods, leading to 15 heart rate scores during the 3 s interval. We then expressed these 15 heart scores as a percentage of the BPM of the baseline. Percentage scores were introduced to standardize the differing absolute BPM variations of individual participants and thus enable comparison between individuals and groups.

### Statistical Analyses

In order to obtain our intra-subject measures, two types of odors and fragrances were distinguished on the basis of each participant’s own ratings: *pleasant* (two highest hedonicity scores) and *unpleasant* (two lowest hedonicity scores). We also performed correlations between the mean pleasantness rating of each odorant stimulus corresponding to a given hedonic order (1: least liked odorant to 32: most liked odorant) across individuals and the strength of its corresponding physiological response (heart rate or electrodermal response).

We computed a mixed model analysis of variance (ANOVA), with pleasantness (2: *pleasant, unpleasant*) as the within-subject repeated factor and group (2: *Odor, Fragrance*) as the between-subject factor to analyze subjective ratings, heart rate, electrodermal response, and respiratory parameters.

In the case of facial muscle activity, a Time factor (five: *0–1,1–2,2–3,3–4,4–5 s*) was added to account for the temporal evolution of the signal, decomposed in five 1 s time intervals. We tested the significance of paired comparisons between experimental conditions, using Tukey *post hoc* comparisons (PHCs). All tests were performed by using STATISTICA 12^2^.

## Results

### Subjective Ratings

**FIGURE 1:**
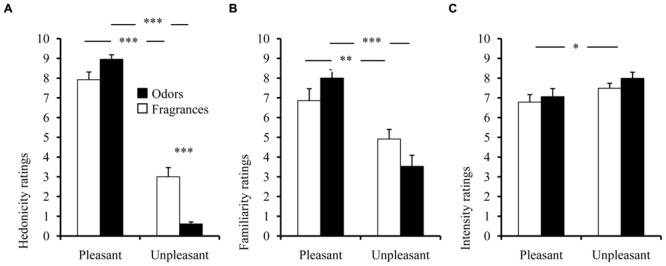
**Mean subjective ratings of **(A)** hedonicity, **(B)** familiarity, and **(C)** intensity of *Odors* and *Fragrances* according to pleasantness.** Vertical bars denote standard errors to the mean. Significance levels for pleasantness effect: n.s.: not significant, *p* > 0.05; ^∗^*p* ≤ 0.05; ^∗∗^*p* ≤ 0.01; ^∗∗∗^*p* ≤ 0.001.

The analysis performed on hedonicity, familiarity, and intensity ratings revealed a main effect of pleasantness on these three indicators [*F*(1,35) = 839.03, *p* < 0.001, $\eta_{s}^{2}$ = 0.96; *F*(1,35) = 77.98 *p* < 0.001, η^2^ = 0.69; and *F*(1,35) = 7.28, *p* = 0.011, η^2^ = 0.17, respectively]. Pleasant stimuli (*odors* and *fragrances*) were systematically evaluated as being more pleasant (average: 8.44) than unpleasant stimuli (1.81), confirming that a clear hedonic distinction was made (**Figure 1A**) based on each participant’s own evaluation. Pleasant stimuli were also perceived as being more familiar (7.43) than unpleasant stimuli (4.22; **Figure 1B**). The group × pleasantness interaction was significant for both hedonic and familiarity ratings [*F*(1,35) = 55.92, *p* < 0.001, η^2^ = 0.61; *F*(1,35) = 12.12, *p* = 0.001, η^2^ = 0.26], revealing a more pronounced hedonic distinction according to pleasantness for *Odors* (PHC *p* < 0.001 for hedonicity and familiarity) compared with *Fragrances* (PHCs *p* < 0.003 for hedonicity and familiarity), since unpleasant *Odors* were rated lower than unpleasant *Fragrances* (**Figure 1A**; PHC, *p* < 0.001). This interaction was not significant for intensity ratings [*F*(1,35) = 0.14, *p* = 0.709, n.s., η^2^ = 0.004; **Figure 1C**], indicating that unpleasant olfactory stimuli were more intense (7.47) than pleasant stimuli (6.92), regardless of the pleasantness spectrum (*Odors* or *Fragrances*).

### Peripheral Physiology

**FIGURE 2:**
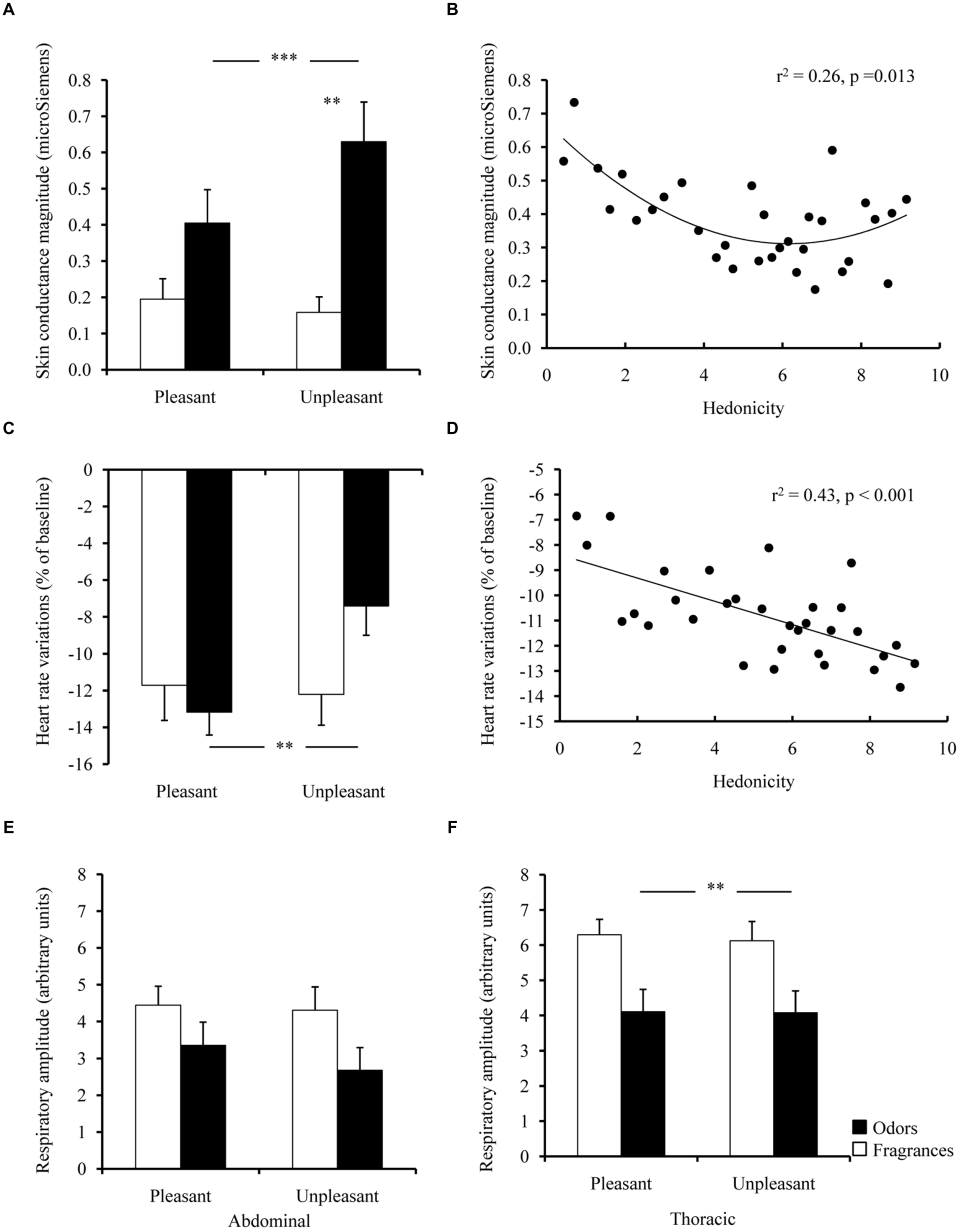
**Peripheral physiology. (A)** Skin conductance, **(C)** heart rate variation (BPM; 5–8 s after stimulus presentation), and **(E)** abdominal and **(F)** thoracic respiratory amplitudes for the intra-individually determined pleasant and unpleasant *Odors* and *Fragrances*. Significant correlations between mean ratings for all odors corresponding to a given hedonic order across individuals **(B)** skin conductance responses and **(D)** heart rate variations. In the graph abscissa, odors pleasantness ratings are arranged from those of least liked odors (corresponding to hedonic order 1), to those of most liked odors (corresponding to hedonic order 32). Vertical bars denote standard errors to the mean. Significance levels for pleasantness effect: n.s.: not significant, *p* > 0.05; ^∗^*p* ≤ 0.05; ^∗∗^*p* ≤ 0.01; ^∗∗∗^*p* ≤ 0.001.

Group × pleasantness interactions were also observed for both peripheral physiological measures, i.e., electrodermal activity and heart rate [*F*(1,35) = 5.75, *p* = 0.022, η^2^ = 0.14; *F*(1,35) = 7.33, *p* = 0.010, η^2^ = 0.17, respectively]. Unpleasant *Odors* elicited stronger SCRs than did pleasant *Odors* (PHC, *p* = 0.033) and unpleasant *Fragrances* (PHC, *p* < 0.001; **Figure 2A**).

Unpleasant *Odors* also specifically induced a weaker heart deceleration than did pleasant *Odors* (PHC, *p* = 0.007), unlike *Fragrances* in which this effect was not significant (**Figure 2C**).

Since both interactions were significant, we performed separate regression analyses between mean hedonicity ratings and SCRs or heart rate, for *Odors* and *Fragrances*, respectively. A significant U-shaped quadratic correlation was found for *Odors* on the SCRs only (*r*^2^ = 0.26, *p* = 0.013), with higher SCRs in response to *Odors* on the extremes of the valence spectrum (very unpleasant or very pleasant), but lower responses to (neutral) *Odors* in the middle of the spectrum (**Figure 2B**). These results were confirmed by a supplementary statistical analysis conducted on electrodermal responses to *Odors*. We conducted a repeated measures ANOVA with pleasantness as **three-level** within-subject repeated factor, in which we took into account a third type of neutral *Odors* (two hedonicity scores located around the median score), in addition to pleasant and unpleasant ones. This analysis revealed a main pleasantness effect [*F*(2,34) = 8.31, *p* = 0.001, η^2^ = 0.33]. A subsequent *post hoc* planned quadratic comparison was performed, with weights of 1, –2, and 1 assigned to pleasant, neutral and unpleasant *Odors*, respectively. This planned comparison was significant [*F*(1,17) = 13.47, *p* = 0.002], confirming that lower SCRs were elicited in response to neutral *Odors* compared to pleasant and unpleasant ones.

In addition, *Odor*-induced heart rate variations correlated negatively with hedonic scores (*r*^2^ = 0.43, *p* < 0.001; **Figure 2D**). However, no significant correlations with *Fragrance* hedonicity ratings were found for either fragrance-induced SCRs or heart rate variations.

Finally, we examined the effects of stimulus pleasantness on respiratory control measures to rule out any confounds that could cause differences at the physiological level. No significant effects of stimulus pleasantness were found on any of the respiratory control measures [*F*(1,35) = 2.96, *p* = 0.094, n.s., η^2^ = 0.03, and *F*(1,35) = 0.27, *p* = 0.600, n.s., η^2^ = 0.01, for abdominal and thoracic respirations, respectively; **Figures 2E,F**], although the general thoracic respiratory amplitude was higher in the *Fragrance* group [*F*(1,35) = 7.52, *p* = 0.001, η^2^ = 0.18].

### Facial Muscle Activity

In general, *Odors* elicited a much stronger expressive activity than did *Fragrances* [main group effects: *F*(1,33) = 4.74, *p* = 0.037, η^2^ = 0.3, and *F*(1,33) = 8.75, *p* = 0.006, η^2^ = 0.21, for corrugator and zygomaticus, respectively]. We found a significant triple Time × Pleasantness × Group interaction for corrugator activity [*F*(4,132) = 2.45, *p* = 0.050, η^2^ = 0.07]. In order to examine these effects in more detail, we performed two separate secondary ANOVAs on corrugator activity, where Time (5: corresponding to 5 s × 1 s windows) was introduced as a multiple dependent variable and pleasantness (2) as a within-subject factor for *Odors* and *Fragrances* separately, since muscular activity shows a sequential evolution (see Delplanque et al., 2009).

**FIGURE 3:**
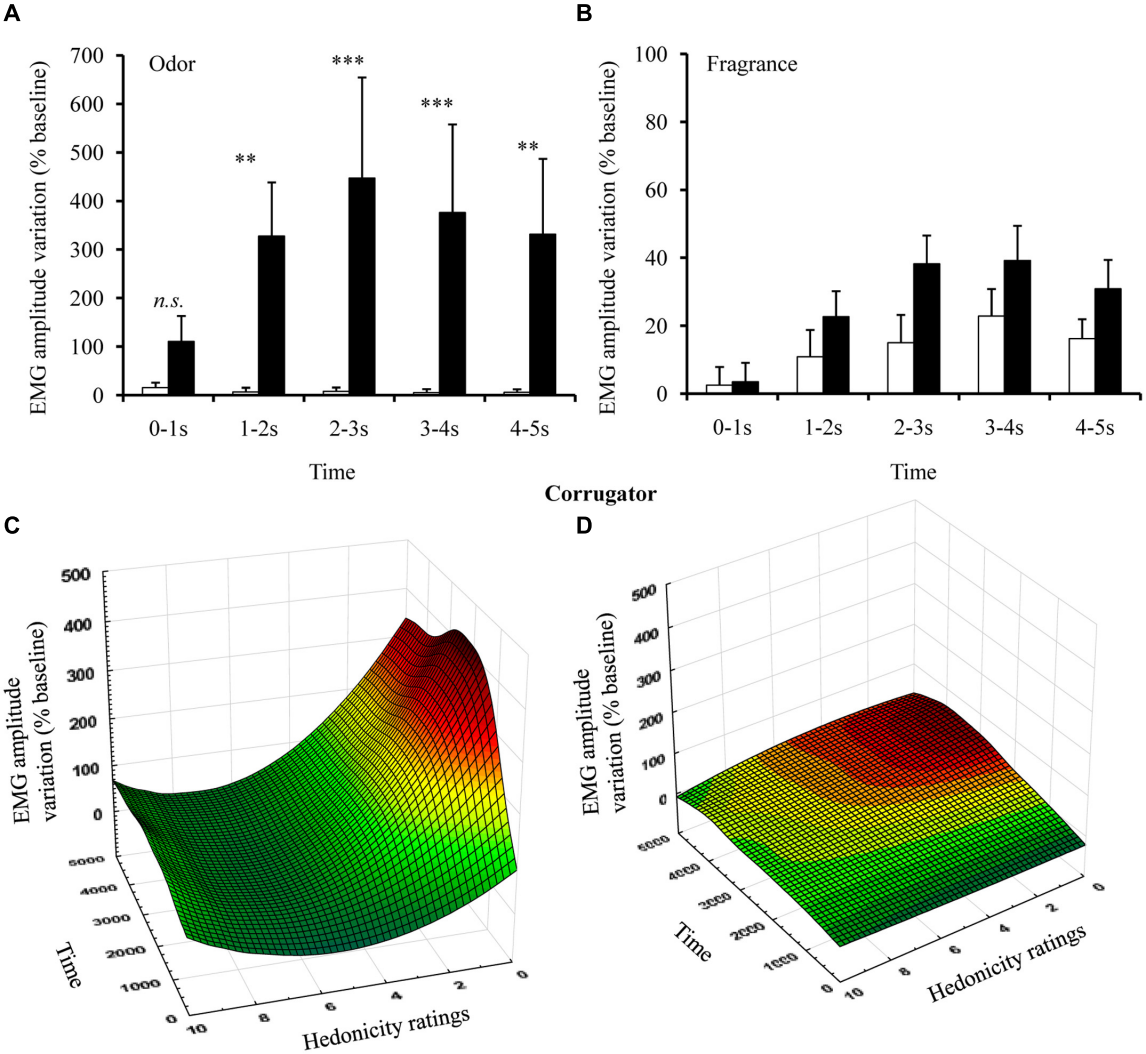
**Corrugator muscle activity. (A)** *Fragrance*- and **(C)** *Odor*-related corrugator activities (EMG; % of baseline) for the intra-individually determined pleasant and unpleasant odors. **(B)** *Fragrance*- and **(D)** *Odor*-related corrugator activity changes (EMG; % of baseline) as a function of time (ms) and hedonicity for all stimuli. Vertical bars denote standard errors to the mean. Significance levels for pleasantness effect: n.s.: not significant, *p* > 0.05; ^∗^*p* ≤ 0.05; ^∗∗^*p* ≤ 0.01; ^∗∗∗^*p* ≤ 0.001.

These analyses revealed a Time × Pleasantness interaction in *Odor*-induced corrugator activity [*F*(4,64) = 2.67, *p* = 0.040, η^2^ = 0.14], with an increase in the percentage of muscular activity in response to unpleasant *Odors* as compared with pleasant *Odors* in all time windows except the first one (PHC *p*s ≤ 0.004; **Figure 3A**). For better visualization of the effect, the continuous evolution of corrugator activity was plotted both as a function of time and of hedonicity scores. The resulting 3D plot showed a combined slope increasing across time toward lower hedonic values of *Odors* (**Figure 3B**). *Fragrance*-induced corrugator activity increased both as a function of unpleasantness [*F*(1,17) = 5.19, *p* = 0.036, η^2^ = 0.23] and of time [*F*(4,68) = 9.83, *p* < 0.001, η^2^ = 0.36; **Figure 3C**], although this increase was relatively small compared with that induced by *Odors* (**Figure 3D**).

**FIGURE 4:**
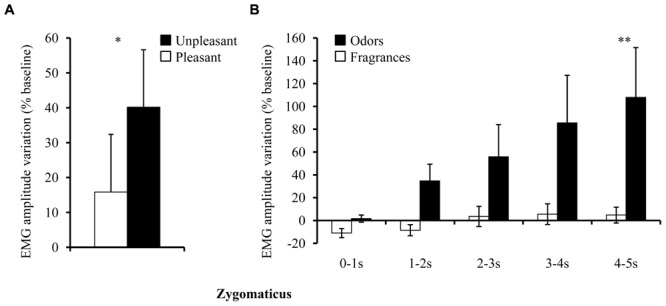
**Zygomaticus muscle activity (EMG; % of baseline) for *Fragrance* and *Odors*. (A)** Main pleasantness effect. **(B)** Main group effect. Vertical bars denote standard errors to the mean. Significance levels for pleasantness effect: n.s.: not significant, *p* > 0.05; ^∗^*p* ≤ 0.05; ^∗∗^*p* ≤ 0.01; ^∗∗∗^*p* ≤ 0.001.

The zygomaticus also showed increased activity in response to both pleasant olfactory stimuli [main pleasantness effect: *F*(1,33) = 6.50, *p* = 0.016, η^2^ = 0.16; **Figure 4A**], although the increase in activity over time was more important for *Odors* than for *Fragrances* [Time × Group interaction: *F*(4,132) = 3.94, *p* = 0.005, η^2^ = 0.11; **Figure 4B**].

## Discussion

In this experiment, we assessed whether subjective, physiological, and expressive indicators differentiate between different ranges of odor and fragrance pleasantness. Our results showed strong distinctions of pleasant and unpleasant *Odors* on the basis of subjective, physiological, and expressive data, in agreement with previous studies (Alaoui-Ismaïli et al., 1997; Bensafi et al., 2002b; Armstrong et al., 2007; Bradley et al., 2008; Delplanque et al., 2009; Sequeira et al., 2009). On the other hand, *Fragrances*, belonging to a more restricted pleasantness spectrum, were mostly differentiated on the basis of their subjective ratings, rather than physiological and expressive indicators.

More specifically, subjective ratings were sensitive to pleasantness, with unpleasant olfactory stimuli perceived as being less familiar and more intense, in line with previous findings (Doty, 1975; Ayabe-Kanamura et al., 1998; Royet et al., 1999; Delplanque et al., 2008), although this distinction between pleasant and unpleasant olfactory stimuli was stronger for *Odors* than for *Fragrances*. At the physiology level, heart rate differentiated between levels of *Odor* pleasantness linearly: the more pleasant the *Odor*, the stronger the decrease, which is in line with previous findings (Soussignan et al., 2005; Delplanque et al., 2009). Electrodermal responses were sensitive to either very pleasant or very unpleasant stimuli. The supplementary analyses performed with an additional category of neutral *Odors* revealed weaker responses to neutral stimuli, suggesting sensitivity to arousing stimulations, in keeping with the U-shaped relation between odor hedonicity and arousal (Doty, 1975; Bensafi et al., 2002b,c; Winston et al., 2005).

No statistically significant modulation of these two physiological indicators (heart rate, SCR) was observed for *Fragrances*, however, suggesting that the sensitivity of physiological indicators to related odors with a narrow range of pleasantness, such as fragrances, is limited, even although subjective hedonic differentiations were clearly reported by the participants. The fact that no effect of pleasantness was observed in any of the respiratory control measures indicates that it is unlikely that the differences observed at the physiological level could be caused by differential patterns of respiration as a function of odor pleasantness. Finally, pleasantness was also differentiated at the expressive level through corrugator activation and, to a lesser extent, through zygomaticus activation, echoing prior results (Bensafi et al., 2002c; Soussignan et al., 2005; Armstrong et al., 2007; Delplanque et al., 2009). The expressive component responded to both *Odors* and *Fragrances*, although *Fragrance*-related activity was much weaker.

This experiment provided information concerning the ability of classic psychophysiological measures to investigate subtle differences in emotional reaction to olfactory stimuli, as it sheds light on the relation between physiological indicators and subjective ratings when characterizing odors with a wide range of pleasantness versus fragrances with a narrow range of pleasantness. Whereas there were clear differences in physiological reactions to odors that were very different in terms of pleasantness, those differences almost entirely vanished when a particular family of products (i.e., only fragrances) with a restricted range of pleasantness was tested. This does not mean that finding subtle physiological differences in response to a restricted range of products is not possible. Rather, it seems that with classic and easy-to-set-up measures, such subtle differences are unlikely to be observed.

Apart from the technical and methodological constraints, there are clear theoretical reasons to expect such a pattern of results. According to appraisal theories of emotion, e.g., the component process model (Scherer, 1982, 2001), the subjective feeling and the physiological response associated with a specific stimulus (e.g., a given odorant) are separate components whose synchronized modification entails an emotional percept. Although related, subjective feeling and physiological response reflect different components of the emotional response. A modification of the subjective feeling component—which is considered to reflect changes in the other components—will not necessarily entail a difference in the physiological or EMG data, the latter reflecting the expressive component. Our results emphasize the importance of measuring several components of an emotional episode.

On the other hand, the physiological responses observed during an emotional episode should be adapted to the demands of the physical and social environment in order to prepare the individual for action (Frijda, 1987; Sander et al., 2005). Similar to emotional cues triggering adaptive behaviors in reaction to environmental events, olfactory stimuli modulate motivational states in a powerful fashion through their relevance, for example, when malodors induce avoidance reactions through the elicitation of profound aversion or disgust (Royet et al., 2001; Gottfried et al., 2002; Anderson et al., 2003). Olfactory stimuli are thus prone to inducing behavioral adaptations to changes in the environment (Pause et al., 2003), resulting in approach or avoid action tendencies (Frijda, 1987). Olfactory stimuli can even be involved in more complex functions, classified as adaptive behaviors for survival: ingestion, hazard avoidance, social communication, and emotional contagion (see Stevenson, 2010 for a review).

Characterizing consumer preferences by objective physiological and/or EMG measures is a goal that many industries would currently like to attain. These measured responses should be able to differentiate among odors that evoke representations linked to different functions of olfaction (Stevenson, 2010), scattered along a wide pleasantness spectrum. In contrast, it is unlikely that the physiological system would respond differentially when the range of pleasantness is narrow, as is the case with fragrances.

Such subtle differences are well characterized by subjective appreciations, as previous evidence suggested that odor-elicited feelings are complex and varied (Chrea et al., 2009). Aside from the utilitarian functions they embody, odor-borne feelings may also be related to more elaborate forms of hedonic appreciation, such as complex esthetic feelings experienced with music (Zentner et al., 2008; Trost et al., 2012). Odor-borne feelings can be accurately described by specific semantic scales (Chrea et al., 2009; Ferdenzi et al., 2011, 2013; Delplanque et al., 2012), which are a reliable tool for the discrimination of products with similar liking scores such as fragrances or flavored products (Porcherot et al., 2010). In the domain of fragrances, differences in ratings of liking have been found when the same fragrances are rated with or without brand labels (Moskowitz, 1979), an effect commonly observed in food perception (Spinelli et al., 2015). Therefore, an important dimension to consider when it comes to fragrances—in particular, fine perfumes—is luxury because of its ecological occurrence in brand information. A luxurious qualification confers additional value and satisfaction to a given product, as well as supplementary information about its source, yet it may not reflect urgent necessities (Kapferer, 1997; Megehee and Spake, 2012) or differential survival-related functions (e.g., they would all be related to well-being; see Stevenson, 2010). It would thus be interesting to investigate the extent to which self-reported and psychophysiological measures could be influenced by information regarding the luxurious character of a fine perfume. This could be done by presenting the same group of participants with fragrances, with and without the corresponding labels, truthful or not, on different days. Aside from liking, the rewarding sensation experienced during any agreeable sensory stimulation also includes a “wanting” component, which translates into motivation to invest effort in order to obtain such a reward (Berridge and Robinson, 1998; Pool et al., 2015). By measuring, for example, the willingness to pay for a specific product, the wanting component would allow a more complete picture of fragrance-based elicited reward and would perhaps enhance the discriminative power of subjective measures for similarly pleasant products.

## Conclusion

In summary, this study shows that emotions elicited by odors that display a wide range of reported pleasantness can be distinguished by both subjective feeling and physiological indicators. These physiological differences almost entirely vanish when odorants belong to a much more restricted pleasantness range, even though the subjective feelings still differ. This work contributes to the literature on emotions by emphasizing the multi-componential nature of emotion and the importance of considering several components when studying olfactory-induced emotions. Finally, our results address the current trend found in many industries to characterize consumer behavior by using physiological measures. Although differences can be expected in response to heterogeneous products in terms of pleasantness, physiological measures appear to show limited sensitivity in distinguishing among similarly pleasant products.

## Conflict of Interest Statement

The authors declare that the research was conducted in the absence of any commercial or financial relationships that could be construed as a potential conflict of interest.

## Footnotes

1 http://www.biosemi.com/faq/cms&drl.htm

2 http://www.statsoft.com

## References

[B1] Adolph D. Schlösser S. Hawighorst M. Pause B. M. (2010). Chemosensory signals of competition increase the skin conductance response in humans. *Physiol. Behav.* 101 666–671. 10.1016/j.physbeh.2010.08.004 20708023

[B2] Alaoui-Ismaïli O. Robin O. Rada H. Dittmar A. Vernet-Maury E. (1997). Basic emotions evoked by odorants: comparison between autonomic responses and self-evaluation. *Physiol. Behav.* 62 713–720. 10.1016/S0031-9384(97)90016-0 9284489

[B3] Anderson A. K. Christoff K. Stappen I. Panitz D. Ghahremani D. G. Glover G. H. (2003). Dissociated neural representations of intensity and valence in human olfaction. *Nat. Neurosci.* 6 196–202. 10.1038/nn1001 12536208

[B4] Armstrong J. E. Hutchinson I. Laing D. G. Jinks A. L. (2007). Facial electromyography: responses of children to odor and taste stimuli. *Chem. Senses* 32 611–621. 10.1093/chemse/bjm029 17510090

[B5] Ayabe-Kanamura S. Saito S. Distel H. Martínez-gómez M. Hudson R. (1998). Differences and similarities in the perception of everyday odors. *Chem. Senses* 23 31–38. 10.1093/chemse/23.1.31 9530967

[B6] Bensafi M. Rinck F. Schaal B. Rouby C. (2007). Verbal cues modulate hedonic perception of odors in 5-year-old children as well as in adults. *Chem. Senses* 32 855–862. 10.1093/chemse/bjm055 17728278

[B7] Bensafi M. Rouby C. Farget V. Vigouroux M. Holley A. (2002a). Asymmetry of pleasant vs. unpleasant odor processing during affective judgment in humans. *Neurosci. Lett.* 328 309–313. 10.1016/S0304-3940(02)00548-7 12147332

[B8] Bensafi M. Rouby C. Farget V. Bertrand B. Vigouroux M. Holley A. (2002b). Autonomic nervous system responses to odours: the role of pleasantness and arousal. *Chem. Senses* 27 703–709. 10.1093/chemse/27.8.703 12379594

[B9] Bensafi M. Rouby C. Farget V. Bertrand B. Vigouroux M. Holley A. (2002c). Psychophysiological correlates of affects in human olfaction. Correlats neurophysiologiques des états affectifs déclenchés par les odeurs chez l’ homme. *Neurophysiol. Clin.* 32 326–332. 12490330 10.1016/s0987-7053(02)00339-8

[B10] Berridge K. C. Robinson T. E. (1998). What is the role of dopamine in reward: hedonic impact, reward learning, or incentive salience? *Brain Res. Rev.* 28 309–369. 10.1016/S0165-0173(98)00019-8 9858756

[B11] Bradley M. M. Miccoli L. Escrig M. A. Lang P. J. (2008). The pupil as a measure of emotional arousal and autonomic activation. *Psychophysiology* 45 602–607. 10.1111/j.1469-8986.2008.00654.x 18282202 PMC3612940

[B12] Carmichael S. T. Clugnet M. C. Price J. L. (1994). Central olfactory connections in the macaque monkey. *J. Comp. Neurol.* 346 403–434. 10.1002/cne.903460306 7527806

[B13] Chrea C. Grandjean D. Delplanque S. Cayeux I. Le Calvé B. Aymard L. (2009). Mapping the semantic space for the subjective experience of emotional responses to odors. *Chem. Senses* 34 49–62. 10.1093/chemse/bjn052 18786964

[B14] Coppin G. Delplanque S. Cayeux I. Porcherot C. Sander D. (2010). I’m no longer torn after choice: how explicit choices implicitly shape preferences of odors. *Psychol. Sci.* 21 489–493. 10.1177/0956797610364115 20424088

[B15] Coppin G. Delplanque S. Porcherot C. Cayeux I. Sander D. (2012). When flexibility is stable: implicit long-term shaping of olfactory preferences. *PLoS ONE* 7:e37857 10.1371/journal.pone.0037857 PMC3380896 22761661

[B16] Coppin G. Sander D. (2011). “The flexibility of chemosensory preferences,” in *The Neuroscience of Preference and Choice* eds Dolan R. J. Sharot T. (Amsterdam: Elsevier Publishing) 257–275.

[B17] Croy I. Negoias S. Novakova L. Landis B. N. Hummel T. (2012). Learning about the functions of the olfactory system from people without a sense of smell. *PLoS ONE* 7:e33365 10.1371/journal.pone.0033365 PMC3310072 22457756

[B18] Dawson M. E. Schell A. M. Filion D. L. (1990). “The electrodermal response system,” in *Principles of Psychophysiology: Physical, Social and Inferential Elements* eds Cacioppo J. T. Tassinary L. G. (Cambridge: Cambridge University Press) 295–324.

[B19] de Araujo I. E. T. Rolls E. T. Velazco M.-I. Margot C. Cayeux I. (2005). Cognitive modulation of olfactory processing. *Neuron* 46 671–679. 10.1016/j.neuron.2005.04.021 15944134

[B20] de Wied M. van Boxtel A. Zaalberg R. Goudena P. P. Matthys W. (2006). Facial EMG responses to dynamic emotional facial expressions in boys with disruptive behavior disorders. *J. Psychiatr. Res.* 40 112–121. 10.1016/j.jpsychires.2005.08.003 16176819

[B21] Degel J. Piper D. Köster E. P. (2001). Implicit learning and implicit memory for odors: the influence of odor identification and retention time. *Chem. Senses* 26 267–280. 10.1093/chemse/26.3.267 11287387

[B22] Delplanque S. Chrea C. Grandjean D. Ferdenzi C. Cayeux I. Porcherot C. (2012). How to map the affective semantic space of scents. *Cogn. Emot.* 26 885–898. 10.1080/02699931.2011.628301 22348313

[B23] Delplanque S. Coppin G. Bloesch L. Cayeux I. Sander D. (2015). The mere exposure effect depends on an odor’s initial pleasantness. *Front. Psychol.* 6:911 10.3389/fpsyg.2015.00920 PMC4490210 26191021

[B24] Delplanque S. Grandjean D. Chrea C. Aymard L. Cayeux I. Le Calvé B. (2008). Emotional processing of odors: evidence for a nonlinear relation between pleasantness and familiarity evaluations. *Chem. Senses* 33 469–479. 10.1093/chemse/bjn014 18403383

[B25] Delplanque S. Grandjean D. Chrea C. Coppin G. Aymard L. Cayeux I. (2009). Sequential unfolding of novelty and pleasantness appraisals of odors: evidence from facial electromyography and autonomic reactions. *Emotion* 9 316–328. 10.1037/a0015369 19485609

[B26] Doty R. L. (1975). An examination of relationships between the pleasantness, intensity, and concentration of 10 odorous stimuli. *Percept. Psychophys.* 17 492–496. 10.3758/BF03203300

[B27] Edelberg R. (1972). Electrodermal recovery rate, goal orientation and aversion. *Psychophysiology* 9 512–520. 10.1111/j.1469-8986.1972.tb01805.x 5075582

[B28] Ferdenzi C. Delplanque S. Barbosa P. Court K. Guinard J.-X. Guo T. (2013). Affective semantic space of scents. Towards a universal scale to measure self-reported odor-related feelings. *Food Qual. Prefer.* 30 128–138. 10.1016/j.foodqual.2013.04.010

[B29] Ferdenzi C. Schirmer A. Roberts S. C. Delplanque S. Porcherot C. Cayeux I. (2011). Affective dimensions of odor perception: a comparison between Swiss, British, and Singaporean populations. *Emotion* 11 1168–1181. 10.1037/a0022853 21534667

[B30] Fridlung A. J. Cacioppo J. T. (1986). Guidelines for human electromygraphic research. *Psychophysiology* 23 567–589. 10.1111/j.1469-8986.1986.tb00676.x 3809364

[B31] Frijda N. H. (1987). Emotion, cognitive structure, and action tendency. *Cogn. Emot.* 1 115–143. 10.1080/02699938708408043

[B32] Gelstein S. Yeshurun Y. Rozenkrantz L. Shushan S. Frumin I. Roth Y. (2011). Human tears contain a chemosignal. *Science* 331 226–230. 10.1126/science.1198331 21212322

[B33] Gottfried J. A. O’Doherty J. P. Dolan R. J. (2002). Appetitive and aversive olfactory learning in humans studied using event-related functional magnetic resonance imaging. *J. Neurosci.* 22 10829–10837. 12486176 10.1523/JNEUROSCI.22-24-10829.2002 PMC6758414

[B34] Grabenhorst F. Rolls E. T. Margot C. da Silva M. A. A. P. Velazco M. I. (2007). How pleasant and unpleasant stimuli combine in different brain regions: odor mixtures. *J. Neurosci.* 27 13532–13540. 10.1523/JNEUROSCI.3337-07.2007 18057211 PMC6673088

[B35] Herz R. S. (2003). The effect of verbal context on olfactory perception. *J. Exp. Psychol. Gen.* 132 595–606. 10.1037/0096-3445.132.4.595 14640850

[B36] Herz R. S. Eliassen J. Beland S. Souza T. (2004a). Neuroimaging evidence for the emotional potency of odor-evoked memory. *Neuropsychologia* 42 371–378. 10.1016/j.neuropsychologia.2003.08.009 14670575

[B37] Herz R. S. Schankler C. Beland S. (2004b). Olfaction, emotion and associative learning: effects on motivated behavior. *Motiv. Emot.* 28 363–383. 10.1007/s11031-004-2389-x

[B38] Herz R. S. Von Clef J. (2001). The influence of verbal labeling on the perception of odors: evidence for olfactory illusions? *Perception* 30 381–391. 10.1068/p3179 11374206

[B39] Howard J. D. Plailly J. Grueschow M. Haynes J.-D. Gottfried J. A. (2009). Odor quality coding and categorization in human posterior piriform cortex. *Nat. Neurosci.* 12 932–938. 10.1038/nn.2324 19483688 PMC2834563

[B40] Hummel T. Nordin S. (2005). Olfactory disorders and their consequences for quality of life. *Acta Otolaryngol.* 125 116–121. 10.1080/00016480410022787 15880938

[B41] Jung J. Hudry J. Ryvlin P. Royet J.-P. Bertrand O. Lachaux J.-P. (2006). Functional significance of olfactory-induced oscillations in the human amygdala. *Cereb. Cortex* 16 1–8. 10.1093/cercor/bhi090 15829732

[B42] Kapferer J.-N. (1997). Managing luxury brands. *J. Brand Manag.* 4 251–259. 10.1057/bm.1997.4

[B43] Keller A. Malaspina D. (2013). Hidden consequences of olfactory dysfunction: a patient report series. *BMC Ear Nose Throat Disord.* 13:8 10.1186/1472-6815-13-8 PMC3733708 23875929

[B44] Keller A. Zhuang H. Chi Q. Vosshall L. B. Matsunami H. (2007). Genetic variation in a human odorant receptor alters odour perception. *Nature* 449 468–472. 10.1038/nature06162 17873857

[B45] Landis B. N. Stow N. W. Lacroix J.-S. Hugentobler M. Hummel T. (2009). Olfactory disorders: the patients’ view. *Rhinology* 47 454–459. 10.4193/Rhin08.174 19936376

[B46] Le Guérer A. (1994). *Scent: The Mysterious and Essential Powers of Smell*. New York: Kodansha America.

[B47] Leppanen J. M. Hietanen J. K. (2003). Affect and face perception: odors modulate the recognition advantage of happy faces. *Emotion* 3 315–326. 10.1037/1528-3542.3.4.315 14674826

[B48] Li W. Howard J. D. Gottfried J. A. (2010). Disruption of odour quality coding in piriform cortex mediates olfactory deficits in Alzheimer’s disease. *Brain* 133 2714–2726. 10.1093/brain/awq209 20724290 PMC2948816

[B49] Li W. Moallem I. Paller K. A. Gottfried J. A. (2007). Subliminal smells can guide social preferences. *Psycholog. Sci.* 18 1044–1049. 10.1111/j.1467-9280.2007.02023.x 18031410

[B50] Megehee C. M. Spake D. F. (2012). Consumer enactments of archetypes using luxury brands. *J. Business Res.* 65 1434–1442. 10.1016/j.jbusres.2011.10.009

[B51] Mohanty A. Gottfried J. A. (2013). “Examining emotion perception and elicitation via olfaction,” in *The Cambridge Handbook of Human Affective Neuroscience* eds Armony J. L. Vuilleumier P. (Cambridge: Cambridge University Press) 241–264.

[B52] Moskowitz H. R. (1979). “An analysis of factors which influence sensory hedonics,” in *Preference Behaviour and Chemoreception* ed. Kroeze J. H. A. (London: Information Retrieval Ltd.) 131–144.

[B53] Pause B. M. Adolph D. Prehn-Kristensen A. Ferstl R. (2009). Startle response potentiation to chemosensory anxiety signals in socially anxious individuals. *Int. J. Psychophysiol.* 74 88–92. 10.1016/j.ijpsycho.2009.07.008 19666058

[B54] Pause B. M. Raack N. Sojka B. Göder R. Aldenhoff J. B. Ferstl R. (2003). Convergent and divergent effects of odors and emotions in depression. *Psychophysiology* 40 209–225. 10.1111/1469-8986.00023 12820862

[B55] Pool E. Brosch T. Delplanque S. Sander D. (2015). Stress increases cue-triggered “Wanting” for sweet reward in humans. *J. Exp. Psychol.* 41 128–136. 10.1037/xan0000052 25734754

[B56] Porcherot C. Delplanque S. Raviot-Derrien S. Le Calvé B. Chrea C. Gaudreau N. (2010). How do you feel when you smell this? Optimization of a verbal measurement of odor-elicited emotions. *Food Qual. Prefer.* 21 938–947. 10.1016/j.foodqual.2010.03.012

[B57] Rétiveau A. N. Chambers E. Milliken G. A. (2004). Common and specific effects of fine fragrances on the mood of women. *J. Sens. Stud.* 19 373–394. 10.1111/j.1745-459x.2004.102803.x

[B58] Royet J.-P. Hudry J. Zald D. H. Godinot D. Grégoire M. C. Lavenne F. (2001). Functional neuroanatomy of different olfactory judgments. *Neuroimage* 13 506–519. 10.1006/nimg.2000.0704 11170816

[B59] Royet J.-P. Koenig O. Gregoire M.-C. Cinotti L. Lavenne F. Le Bars D. (1999). Functional anatomy of perceptual and semantic processing for odors. *J. Cogn. Neurosci.* 11 94–109. 10.1162/089892999563166 9950717

[B60] Sander D. Grandjean D. Scherer K. R. (2005). A systems approach to appraisal mechanisms in emotion. *Neural Netw.* 18 317–352. 10.1016/j.neunet.2005.03.001 15936172

[B61] Savic I. Gulyás B. Berglund H. (2002). Odorant differentiated pattern of cerebral activation: comparison of acetone and vanillin. *Hum. Brain Mapp.* 17 17–27. 10.1002/hbm.10045 12203685 PMC6871790

[B62] Scherer K. R. (1982). Emotion as a process: function, origin and regulation. *Soc. Sci. Inf.* 21 555–570. 10.1177/053901882021004004

[B63] Scherer K. R. (2001). “Appraisal considered as a Process of Multilevel Sequential Checking,” in *Appraisal Processes in Emotion: Theory, Methods, Research* eds Scherer K. R. Schorr A. Johnstone T. (New York: Oxford University Press) 92–120.

[B64] Sequeira H. Hot P. Silvert L. Delplanque S. (2009). Electrical autonomic correlates of emotion. *Int. J. Psychophysiol.* 71 50–56. 10.1016/j.ijpsycho.2008.07.009 18723054

[B65] Smeets M. Dalton P. (2002). Perceived odor and irritation of isopropanol: a comparison between naïve controls and occupationally exposed workers. *Int. Arch. Occup. Environ. Health* 75 541–548. 10.1007/s00420-002-0364-y 12373316

[B66] Soussignan R. Ehrlé N. Henry A. Schaal B. Bakchine S. (2005). Dissociation of emotional processes in response to visual and olfactory stimuli following frontotemporal damage. *Neurocase* 11 114–128. 10.1080/13554790590922513 16036466

[B67] Spinelli S. Masi C. Zoboli G. P. Prescott J. Monteleone E. (2015). Emotional responses to branded and unbranded foods. *Food Qual. Prefer.* 42 1–11. 10.1016/j.foodqual.2014.12.009

[B68] Stevenson R. J. (2010). An initial evaluation of the functions of human olfaction. *Chem. Senses* 35 3–20. 10.1093/chemse/bjp083 19942579

[B69] Trost W. Ethofer T. Zentner M. Vuilleumier P. (2012). Mapping aesthetic musical emotions in the brain. *Cereb. Cortex* 22 2769–2783. 10.1093/cercor/bhr353 22178712 PMC3491764

[B70] Warrenburg S. (2005). Effects of fragrance on emotions: moods and physiology. *Chem. Senses* 30(Suppl. 1) i248–i249. 10.1093/chemse/bjh208 15738139

[B71] Winston J. S. Gottfried J. A. Kilner J. M. Dolan R. J. (2005). Integrated neural representations of odor intensity and affective valence in human amygdala. *J. Neurosci.* 25 8903–8907. 10.1523/JNEUROSCI.1569-05.2005 16192380 PMC6725588

[B72] Zelano C. Montag J. Johnson B. N. Khan R. M. Sobel N. (2007). Dissociated representations of irritation and valence in human primary olfactory cortex. *J. Neurophysiol.* 97 1969–1976. 10.1152/jn.01122.2006 17215504

[B73] Zentner M. Grandjean D. Scherer K. R. (2008). Emotions evoked by the sound of music: characterization, classification, and measurement. *Emotion* 8 494–521. 10.1037/1528-3542.8.4.494 18729581

